# Data on the effect of heat and other technical variables on the detection of microRNAs in human serum

**DOI:** 10.1016/j.dib.2019.103750

**Published:** 2019-03-07

**Authors:** Luísa Camacho, Patricia Porter-Gill, Camila S. Silva

**Affiliations:** Division of Biochemical Toxicology, National Center for Toxicological Research, Food and Drug Administration, Jefferson, AR, USA

## Abstract

Data are presented on the number and levels of 384 microRNAs (miRNAs) quantified by reverse-transcription real-time quantitative polymerase chain reaction (RT-qPCR) in human serum analyzed under different experimental conditions. The technical variables tested were 1) heating of the serum samples at 60 °C for 120 minutes prior to RNA extraction *versus* no heating; 2) RNA extraction using an Exiqon miRCURY RNA Isolation kit for Biofluids *versus* a Systems Biosciences SeraMir Exosome RNA Purification kit; 3) miRNA quantitation by RT-qPCR using an Exiqon SYBR Green Human Panel I *versus* an Applied Biosystems TaqMan Human microRNA Array A.

**Table:** Specifications table

Subject area	*Biology*
More specific subject area	*Quantitation of human serum miRNAs by RT-qPCR*
Type of data	*Table, graph, figure*
How data was acquired	*qPCR using an ABI 7900HT Real-Time PCR System equipped with a TaqMan Low Density Arrays (TLDA) or a 384-well block*
Data format	*Raw and analyzed data*
Experimental factors	*Human serum samples were divided into two equal-volume aliquots; one aliquot of each serum sample was heated at 60 °C for 120 minutes, while the other one was not.*
Experimental features	*RNA was isolated from the heated and non-heated serum samples using an Exiqon miRCURY RNA Isolation kit for Biofluids or a Systems Biosciences SeraMir Exosome RNA Purification kit. cDNA synthesized from the purified RNAs was used as a template to quantify the levels of 384 miRNAs by quantitative real-time polymerase chain reaction (qPCR) using two different platforms (Applied Biosystems TLDA Human microRNA Array A and Exiqon Human Panel I V4.M).*
Data source location	*Jefferson, AR, USA*
Data accessibility	*Data provided with this article*
Related research article	E.M. Kroh, R.K. Parkin, P.S. Mitchell, M. Tewari, Analysis of circulating microRNA biomarkers in plasma and serum using quantitative reverse transcription-PCR (qRT-PCR), Methods. 50 (2010) 298–301. https://doi.org/10.1016/j.ymeth.2010.01.032

**Table:** 

**Value of the data** - Data on the effect that different experimental conditions may have on the detection of human serum miRNA transcripts can be helpful for other researchers to assess how these technical variables may impact the outcome of their studies - Our data contribute to a better understanding of the potential confounding effects that a serum heating step, which may be required prior to the processing of serum samples infected with viruses such as the human immunodeficiency virus (HIV), and RNA purification and quantification methods may have on the quantitation of circulating miRNAs - Our data can be used by other researchers with an interest in circulating miRNAs, including those assessing the potential of miRNAs as sensitive and/or specific clinical biomarkers of disease and toxicity

## 1 Data

**Fig. 1:**
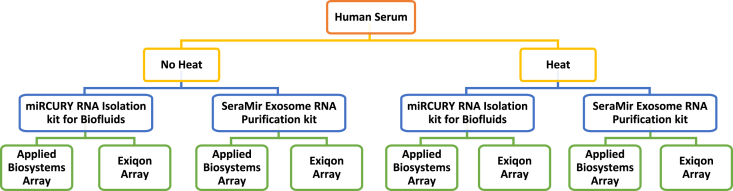
Outline of the experimental conditions tested, which included 1) heating of the serum samples at 60 °C for 120 minutes prior to RNA extraction *versus* no heating; 2) RNA extraction using an Exiqon miRCURY RNA Isolation kit for Biofluids *versus* a Systems Biosciences SeraMir Exosome RNA Purification kit; 3) miRNA quantitation by RT-qPCR using an Exiqon SYBR Green Human Panel I *versus* an Applied Biosystems TaqMan Human microRNA Array A.

**Fig. 2:**
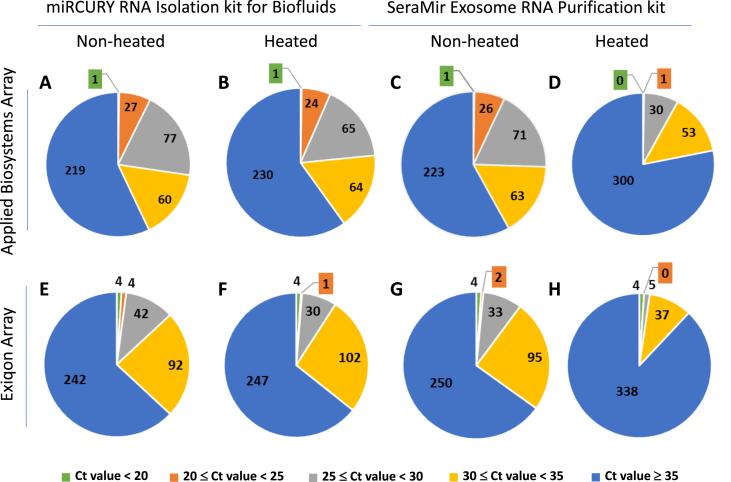
Distribution of mean Ct values of quantified miRNAs across the different experimental conditions tested. **A**, RNA was extracted from non-heated serum samples using a miRCURY RNA Isolation kit for Biofluids and miRNAs were quantified using Applied Biosystems RT-qPCR; **B**, RNA was extracted from heated serum samples using a miRCURY RNA Isolation kit for Biofluids and miRNAs were quantified using Applied Biosystems RT-qPCR; **C**, RNA was extracted from non-heated serum samples using a SeraMir Exosome RNA Purification kit and miRNAs were quantified using Applied Biosystems RT-qPCR; **D**, RNA was extracted from heated serum samples using a SeraMir Exosome RNA Purification kit and miRNAs were quantified using Applied Biosystems RT-qPCR; **E**, RNA was extracted from non-heated serum samples using a miRCURY RNA Isolation kit for Biofluids and miRNAs were quantified using Exiqon RT-qPCR; **F**, RNA was extracted from heated serum samples using a miRCURY RNA Isolation kit for Biofluids and miRNAs were quantified using Exiqon RT-qPCR; **G**, RNA was extracted from non-heated serum samples using a SeraMir Exosome RNA Purification kit and miRNAs were quantified using Exiqon RT-qPCR; **H**, RNA was extracted from heated serum samples using a SeraMir Exosome RNA Purification kit and miRNAs were quantified using Exiqon RT-qPCR.

The data shown in this article provide a comparison of the detection and quantitation of miRNAs in human serum samples that were subjected or not subjected to a heating step prior to RNA purification. Total RNA was extracted from paired non-heated and heated serum samples using a miRCURY RNA Isolation kit for Biofluids (Exiqon) or a SeraMir Exosome RNA Purification kit (Systems Biosciences), and the levels of 384 miRNAs were quantified by RT-qPCR using Human microRNA Array A (Applied Biosystems) or Human Panel I, V4.M (Exiqon). Fig. 1 illustrates the different technical variables assessed. Supplemental File 1 contains the raw qPCR data, which consist of the miRNA threshold cycle (Ct) values detected in each sample. Fig. 2 shows the distribution of the Ct values across the different experimental conditions tested. The Applied Biosystems arrays tended to yield lower Ct values than the Exiqon arrays, likely due to the inclusion of a pre-amplification step of the RT product prior to the qPCR step (Fig. 2A–D *versus* Fig. 2E–H). In the non-heated samples, the source of the RNA did not have a substantial impact on the number of miRNAs detected or on the distribution of their Ct values (Fig. 2A *versus* Fig. 2C, and Fig. 2E *versus* Fig. 2G). In samples derived from RNA extracted using the miRCURY RNA Isolation kit for Biofluids (Exiqon), the pattern was also similar between the non-heated and heated samples (Fig. 2A *versus* Fig. 2B, and Fig. 2E *versus* Fig. 2F), suggesting that the heating step did not destabilize serum miRNAs under these experimental conditions. However, samples derived from RNA extracted using a SeraMir Exosome RNA Purification kit (Systems Biosciences) were affected by the heating step, as illustrated by the overall increase of the Ct values and consequent lower number of miRNA species detected in the heated samples compared to the paired non-heated samples (Fig. 2C *versus* Fig. 2D, and Fig. 2G *versus* Fig. 2H). The effect of heat was observed regardless of the RT-qPCR protocol used (Fig. 2D and H).

## 2 Experimental design, materials, and methods

### 2.1 Source and pre-treatment of the human serum samples

Single donor human serum samples (n = 4) were purchased from Innovative Research (Novi, MI, USA) and divided into two equal-volume aliquots. One aliquot per serum sample was heated at 60 °C for 120 min.

### 2.2 RNA purification

Total RNA, including miRNAs, was isolated from the heated and non-heated serum samples using a miRCURY RNA Isolation kit for Biofluids (Exiqon, Vedbaek, Denmark) or a SeraMir Exosome RNA Purification kit (Systems Biosciences, Mountain View, CA, USA). Two hundred μL of serum were used to extract RNA using the miRCURY RNA Isolation kit. MS2 RNA carrier (Roche Applied Science, Indianapolis, IN, USA) was added to the lysis solution to enhance RNA isolation efficiency. An additional 250 μL aliquot of the same serum samples was used to extract RNA using a SeraMir Exosome RNA Purification kit.

### 2.3 Exiqon RT-qPCR

The Exiqon RT-qPCR procedure was similar to that described previously [1]. Briefly, cDNA was synthesized using a miRCURY LNA Universal cDNA Synthesis Kit II (Exiqon). The RT product was used as a template in the qPCR assays in combination with MicroRNA Ready-to-use PCR Human Panel I V4.M (Exiqon) and ExiLENT SYBR Green master mix (Exiqon). ROX (Invitrogen by Life Technologies, Carlsbad, CA, USA) was added to the master mix as the passive reference dye. The arrays were run in an ABI 7900HT Real-Time PCR System equipped with a 384-well block (Applied Biosystems, Foster City, CA, USA). The qPCR cycling conditions were 95 °C for 10 min and 40 cycles (95 °C for 15 sec and 60 °C for 1 min), followed by a dissociation stage (95 °C for 15 sec, 60 °C for 15 sec, and 95 °C for 15 sec). A melting curve was generated for each assay and yielded a single peak per miRNA (data not shown).

### 2.4 Applied biosystems RT-qPCR

For the Applied Biosystems RT-qPCR assays, RNA was reverse-transcribed using a TaqMan MicroRNA Reverse Transcription kit (Applied Biosystems) and Human Pool A MegaPlex RT primers (Applied Biosystems). The RT product was pre-amplified (12 cycles) with MegaPlex PreAmp primers (Applied Biosystems) and used as a template in the qPCR assays in combination with TaqMan Low Density Human microRNA Array A (Applied Biosystems) and Taqman Universal Master Mix, no AmpErase UNG (Applied Biosystems). The arrays were run in an ABI 7900HT Real-Time PCR System equipped with a TLDA block (Applied Biosystems). The qPCR cycling conditions were 94.5 °C for 10 min and 40 cycles (97 °C for 30 sec and 59.7 °C for 1 min).

### 2.5 RT-qPCR data analysis

The amplification and melting curves were analyzed using Sequence Detection Systems (SDS) software, version 2.4.1 (Applied Biosystems). The Ct values of the four biological replicates per experimental condition were averaged prior to calculating the number of miRNAs detected and the distribution of their Ct values.

## Disclaimer

The views expressed in this manuscript do not necessarily reflect those of the U.S. Food and Drug Administration. The mention of any manufacturers or trade names is only for clarity and does not constitute endorsement.

## Appendix ASupplementary data

Supplemental File 1 Raw data of the RT-qPCR assays included in the current manuscript. Supplemental File 1
